# Moral courage of palliative care nurses and affecting factors

**DOI:** 10.1186/s12904-025-01829-9

**Published:** 2025-07-01

**Authors:** Dilek Yildirim, Vildan Kocatepe, Ayşegül Türkmenoğlu

**Affiliations:** 1 Department of Nursing, Faculty of Health Sciences, Istanbul Aydin University, Florya Campus (Halit Aydın Campus) Inönü Street No: 38 Sefaköy - Kucukcekmece, Istanbul, Turkey; 2 Department of Nursing, Faculty of Health Sciences, Izmir Demokrasi University, Izmir, Turkey; 3 Izmir City Hospital, Izmir, Turkey

**Keywords:** Ethics moral, Courage, Nursing, Palliative care

## Abstract

**Background:**

Moral courage among palliative care nurses plays a critical role in ensuring high-quality care for patients in the final stages of life. This study aimed to assess the level of moral courage among palliative care nurses and identify the factors that influence it.

**Methods:**

This descriptive, cross-sectional study was conducted between September 2023 and September 2024, involving 181 palliative care nurses. Data were collected through an online survey distributed to nurses who voluntarily agreed to participate. The survey included a “Personal Information Form” and the “Nurses’ Moral Courage Scale (NMCS).”

**Results:**

The mean age of participants was 31.82 ± 7.92 years, and their average professional experience was 9.33 ± 9.32 years. Of the nurses, 62.4% were female. The average NMCS score was 82.25 ± 12.21. Nurses with a master’s degree scored significantly higher on the NMCS compared to those with lower educational levels (*p* < 0.001). Additionally, nurses who had chosen their current unit voluntarily (*p* = 0.046), and those who rated their knowledge of healthcare ethics as “excellent” (*p* < 0.001), demonstrated significantly higher moral courage scores. A positive correlation was found between moral courage and age (*r* = 0.153, *p* = 0.040), whereas a negative correlation was observed between moral courage and the average number of night shifts per month (*r* = -0.253, *p* = 0.001).

**Conclusion:**

The findings suggest that palliative care nurses generally exhibit high levels of moral courage. Educational attainment, voluntary selection of the work unit, and perceived competence in healthcare ethics were positively associated with moral courage. Conversely, challenging working conditions—such as high workloads, frequent night shifts, and insufficient institutional support—may hinder nurses’ ethical decision-making by fostering caution and hesitation.

## Introduction

Palliative care is a patient-centered healthcare approach designed to enhance the quality of life for individuals facing life-threatening illnesses. Although modern medicine has made considerable progress in the treatment and management of chronic and terminal conditions, these advancements have simultaneously led to more complex, long-term care needs and have brought forth a wide range of ethical responsibilities for healthcare providers [1–3]. The role of nurses in palliative care goes far beyond the delivery of basic medical care; they serve as key advocates and caregivers who address not only patients’ physical symptoms but also their emotional, psychological, and social needs. By fostering trust, providing empathetic support, and facilitating communication among patients, families, and the healthcare team, nurses are central to ensuring holistic and compassionate end-of-life care [4].

In palliative care settings, nurses frequently navigate complex ethical dilemmas, including upholding patient autonomy, facilitating informed consent, and supporting end-of-life decision-making. These challenges require not only clinical competence but also a strong ethical foundation, as nurses must balance respect for patient preferences with medical realities and institutional constraints [5, 6]. Nurses’ capacity to effectively manage these ethical challenges and the moral courage they exhibit are essential determinants of the quality of care provided to patients. Moral courage is defined as the nurse’s dedication to ethical principles, their willingness to face difficult circumstances, and their resolve to take appropriate action in the best interest of the patient [4, 7, 8].

Developing moral courage is widely acknowledged as a vital approach to mitigating the occurrence of moral distress. Moral courage entails the capacity to steadfastly adhere to ethical principles despite encountering obstacles or unfavorable consequences. Within the nursing profession, it is understood as the ability to act in alignment with established professional ethical codes, empowering nurses to maintain ethical integrity even when confronted with personal risks or adverse outcomes [7, 9, 10]. Research indicates that nurses with high levels of moral courage experience less moral distress [7, 11]. A well-developed sense of moral courage enables nurses to confidently handle challenging circumstances, advocate for professional values, and resist unethical behaviors. Nurses who possess strong moral courage actively protect patients’ rights, make ethically sound decisions, and consistently uphold ethical standards. Conversely, a lack of moral courage may result in ethical compromises, heightened moral distress, and ultimately, a reduction in the quality of patient care [11].

Effective nursing care depends heavily on a supportive work environment. This is especially true for palliative care nurses, who must draw on a high degree of moral courage to navigate the complex challenges and ethical dilemmas inherent in end-of-life care. Such moral resilience not only contributes to greater professional fulfillment but also positively influences the overall quality of patient care [11, 12].

Multiple factors impact the moral courage of palliative care nurses, including their educational background, work environment, professional experience, and access to psychological support systems. Nurses’ understanding of palliative care significantly shapes their ability to make ethical decisions. Research shows that many nurses lack formal training in palliative care, which further complicates their capacity to effectively address the ethical challenges they encounter [13]. Designing educational programs to enhance nurses’ ethical sensitivity can help strengthen their moral courage.

The work environment is another crucial factor affecting nurses’ moral courage. A supportive workplace enhances nurses’ ability to handle ethical dilemmas and positively impacts their moral motivation [14]. Additionally, the presence of professional support systems helps nurses cope with the challenges they encounter. Palliative care nurses frequently face emotionally demanding situations, which can impact their psychological well-being and, consequently, reduce their moral courage [11, 14, 15]. Establishing mechanisms that provide emotional support can facilitate nurses’ ability to manage these challenges more effectively.

The moral courage exhibited by palliative care nurses has a direct impact on the quality of care delivered to patients nearing the end of life. The ethical challenges nurses encounter are influenced by a combination of individual attributes and organizational factors. Enhancing moral courage is essential for enabling nurses to provide care that is both effective and ethically sound. Consequently, fostering their professional growth, improving educational opportunities, and raising awareness of ethical issues represent critical strategies to support this aim.

Although moral courage is essential for nurses, particularly those working in emotionally and ethically complex areas such as palliative care, the existing literature lacks empirical studies specifically focused on this group [16, 17]. Palliative care nurses are uniquely exposed to frequent ethical dilemmas, including end-of-life decisions, patient autonomy, and managing family expectations, all of which require high levels of moral courage. However, little is known about how these nurses experience and develop moral courage within the context of their work [16–19]. This gap highlights the need for research that systematically explores the moral courage of palliative care nurses and the individual and organizational factors that influence it. Therefore, this study seeks to address this gap by evaluating the moral courage levels of palliative care nurses and identifying the factors that affect them.

## Method

### Study design, setting, and sample

This descriptive, cross-sectional study was conducted between September 2023 and September 2024 with the participation of 181 nurses working in palliative care units of hospitals in Turkey. All participants were staff nurses providing direct care to hospitalized patients diagnosed with cancer, neurological diseases, cardiovascular conditions, and other life-limiting illnesses. These nurses were employed in hospital-based palliative care clinics and were not working in primary care settings or nursing homes. In Turkey, the nursing profession is regulated and can be practiced by individuals who graduate from one of the following three types of educational institutions: Health Vocational High School, Associate Degree (two-year college programs), or Bachelor’s Degree (four-year university programs). Regardless of the educational pathway, all graduates obtain the legal right to work as registered nurses after completing their respective programs. Among these nurses, those who have completed a bachelor’s degree are eligible to pursue graduate education such as a master’s degree. In this study, all participants held at least a Health Vocational High School diploma, and some had completed associate or bachelor’s degrees. Nurses who voluntarily agreed to participate in the study, had at least three months of experience working in palliative care, and had internet access were included in the sample. Participants were provided with information about the study, and informed consent was obtained through the initial question of the survey. The entire data collection process took approximately 15 min per participant. To maintain anonymity and confidentiality, email addresses and electronic IP tracking were disabled. A post hoc power analysis was conducted using the G*Power 3.1.9.7 software [20]. Based on the total sample size of 181, the correlation coefficient was calculated as 0.253. With a 5% margin of error (α = 0.05), the power of the study (1-β) was determined to be 0.96, indicating a strong statistical power for correlation analysis.

### Data collection and instruments

In the study, the “Personal Information Form” and the “Nurses’ Moral Courage Scale (NMCS)” were used to collect research data.

### Participant information form

This form was prepared by the researchers based on a review of the relevant literature. It consisted of 16 questions addressing the participating nurses’ sociodemographic characteristics and ethical attributes [7, 9, 10].

### Nurses’ moral courage scale (NMCS-TR)

The “Nurses’ Moral Courage Scale” was developed by Numminen et al. in 2019 to measure nurses’ moral courage [21]. The total possible scores on the scale range from a minimum of 21 to a maximum of 105. Higher scores indicate a stronger adherence to professional ethical and moral principles and a greater commitment to doing what is right for the benefit of the patient. The validity and reliability of NMCS Turkish (NMCS-TR) was conducted by Ayaz & Akkuş in 2023. In terms of construct validity, the original NMCS comprises four subscales and 21 items. The study used confirmatory factor analysis to determine whether the scale conformed to a four-factor structure in the Turkish population. However, the factor distributions showed that the NMCS-TR was unable to distinguish significant differences based on any theoretical structure. Furthermore, the NMCS-TR item distribution differed from that of the original NMCS. Item 11 showed high loading on two factors. Principal components analysis, a form of exploratory factor analysis, was then used to determine the factor structure. The results of this analysis showed that the NMCS-TR had a one-factor structure. The scale’s Cronbach’s alpha coefficient was determined as 0.93, indicating high reliability [22]. The Cronbach alpha coefficient of the scale for this study is 0.931.

### Data analysis

Data analysis was performed using SPSS version 26.0. The socio-demographic characteristics of the participants were analyzed descriptively. To assess the normality of the data distribution, both the Kolmogorov-Smirnov and Shapiro-Wilk tests were applied using multiple normality assessment methods. Results indicated that the data were normally distributed. Descriptive statistics were presented as frequencies, percentages, and means. For comparisons between two groups with normally distributed continuous variables, the Student’s t-test was employed. One-Way Analysis of Variance (ANOVA) was used for comparisons involving more than two groups with normally distributed continuous variables. For comparisons across more than two groups with non-normally distributed continuous variables, the Kruskal-Wallis test was utilized. A p-value of less than 0.05 was considered statistically significant.

### Ethical approval

Ethical approval for the research was obtained from the Social and Human Sciences Ethics Committee of Istanbul Aydin University with the decision number 2023/04 dated 04.05.2023. Moreover, after being informed about the research, all participants consented to participate in the study through the online survey. The research was conducted in accordance with the Declaration of Helsinki.

## Results

The mean age of the palliative care nurses was 31.82 ± 7.92 years, with an average professional experience of 9.33 ± 9.32 years. Nurses had worked in the palliative care unit for an average of 3.00 ± 2.60 years. On average, participants worked 182.85 ± 41.80 h per month and completed 5.98 ± 4.17 night shifts monthly (Table 1).

**Table 1:** Socio-demographic and clinical data of the participants (*n* = 181)

Characteristics	Mean ± SD	Min. -Max.	
**Age (years)**	31.823 ± 7.924	22–52	
**Duration of professional experience (years)**	9.329 ± 9.320	0–35.0	
**Years of working in the palliative care unit**	2.999 ± 2.598	0–17.0	
**Monthly working hours**	182.845 ± 41.797	40–401	
**Number of night shifts per month**	5.983 ± 4.169	0–20	
**Parameters**		**n**	**%**
**Gender**	Female	113	62.4
	Male	68	37.6
**Marital Status**	Married	92	50.8
	Single	89	49.2
**Parenting Status**	Yes	82	45.3
	No	99	54.7
**Educational Level**	Health vocational high school	11	6.1
	Associate degree	10	5.5
	Bachelor’s degree	136	75.1
	Master’s degree	24	13.3
**The preference of choosing one’s work unit**	Yes	67	37.0
	No	114	63.0
**Level of basic knowledge about health ethics**	Insufficient	9	5.0
	Sufficient	82	45.3
	Good	75	41.4
	Excellent	15	8.3
How did you acquire basic knowledge about healthcare ethics? *	Through professional healthcare education	148	81.8
	Through other ethics training (such as courses, continuous education)	108	59.7
**Have you actively participated in any activities and/or development work related to healthcare services ethics at your workplace/organization (e.g.**,** ethics committee)?**	Yes	30	16.6
	No	151	83.4
**Have you encountered situations in your nursing practices where you felt the need to demonstrate moral courage?**	Yes	156	86.2
	No	25	13.8

*Multiple options can be selected

Of the nurses, 62.4% were female, 50.8% were married, and 54.7% reported having no children. The majority (75.1%) held a bachelor’s degree. Additionally, 63% (*n* = 114) of the nurses responded “no” to the question “Did you choose the unit you work in?”. Regarding the acquisition of basic knowledge about healthcare ethics, 81.8% indicated they gained this knowledge through professional healthcare training. Most nurses (83.4%, *n* = 151) reported that they did not actively participate in ethical activities at their workplace, while 86.2% stated that they believed moral courage is necessary in nursing practice and had encountered situations requiring it (Table 1).

The average score on the Nurses’ Moral Courage Scale (NMCS) was 82.25 ± 12.21 (Table 2). When NMCS scores were analyzed according to nurses’ personal and work-related characteristics, no statistically significant differences were observed based on gender, how they acquired healthcare ethics knowledge, active participation in ethical activities at work, or having faced situations requiring moral courage in nursing practice (*p* > 0.05) (Table 3). However, nurses with a master’s degree had significantly higher NMCS scores compared to other educational groups (*p* < 0.001). Nurses who had chosen their work unit scored significantly higher than those who had not (*p* = 0.046). Furthermore, nurses who rated their basic knowledge of healthcare ethics as “excellent” had significantly higher NMCS scores than others (*p* < 0.001) (Table 3).

**Table 2:** Nurses’ moral courage scale scores (*n* = 181)

Scales And Subscales	Mean ± SD	Min-Max*
Total Scores of the Nurses’ Moral Courage Scale	82.254 **±** 12.207	54–105

* Minimum and Maximum score of the scales

**Table 3:** Nurses’ moral courage scale scores according to their personal and work conditions (*n* = 181)

Parameters		Nurses’ Moral Courage Scale
		Mean ± SD
**Gender**	Female	82.477 ± 11.836
	Male	81.882 ± 12.880
**t**		0.317
**p**		0.752
**Educational Level**	Health vocational high school	72.000 ± 11.313
	Associate degree	87.900 ± 16.387
	Bachelor’s degree	80.941 ± 11.212
	Master’s degree	92.041 ± 9.652
**KW**		26.157
**p**		**0.000**
**The preference of choosing one’s work unit**	Yes	84.611 ± 12.592
	No	80.868 ± 11.811
**t**		2.009
**p**		**0.046**
**Level of basic knowledge about health ethics**	Insufficient	71.222 ± 14.981
	Sufficient	80.219 ± 9.751
	Good	83.773 ± 13.365
	Excellent	92.400 ± 8.253
**F**		7.856
**p**		**0.000**
How did you acquire basic knowledge about healthcare ethics? *	Through professional healthcare education	82.500 ± 12,087
	Through other ethics training (such as courses, continuous education)	81.151 ± 12,862
**t**		0.573
**p**		0.585
**Have you actively participated in any activities and/or development work related to healthcare services ethics at your workplace/organization (e.g.**,** ethics committee)?**	Yes	80.500 ± 12.979
	No	82.602 ± 12.062
**t**		− 0.860
**p**		0.390
**Have you encountered situations in your nursing practices where you felt the need to demonstrate moral courage?**	Yes	83.639 ± 12.727
	No	78.600 ± 13.114
**t**		1.779
**p**		0.078

t = t-Test, Z = Mann-Whitney U, KW = Kruskal-Wallis, F = One-way ANOVA; * Multiple options can be selected

A statistically significant positive correlation was found between nurses’ NMCS scores and their average age (*r* = 0.153, *p* = 0.040). Conversely, there was a statistically significant negative correlation between NMCS scores and the average number of night shifts per month (*r* = -0.253, *p* = 0.001) (Table 4).

**Table 4:** Correlation analysis

Variables		1	2	3	4
**1. Nurses’ Moral Courage Scale**	***r***	1			
	***p***	**-**			
**2. Age**	***r***	0.153*	1		
	***p***	**0.040**			
**3. Duration of professional experience (years)**	***r***	0.132	0.954**	1	
	***p***	0.076	**0.000**		
**4. Monthly working hours**	***r***	− 0.059	− 0.105	− 0.132	1
	***p***	0.428	0.159	0.077	
**5. Number of night shifts per month**	***r***	− 0.253**	− 0.325**	− 0.295**	0.105
	***p***	**0.001**	**0.000**	**0.000**	0.159

Note: Pearson’s correlation test. p values in bold indicates statistical significance. Abrreviation: r, correlation coefficient. *p < 0.05; **p < 0.01

## Discussion

The results of the study showed that the moral courage scores of palliative care nurses were high.

In a similar study, nurses’ moral courage was found to be high, measured by both the Nurses Moral Courage Scale and the VAS, and in that study, a higher ethical knowledge base and more self-study or other educational activities were significantly associated with higher moral courage [9]. Moral courage is an effective factor in making ethical decisions, reducing moral distress, and improving the quality of patient care [23]. A study also reported that higher moral courage in nurses was associated with less moral distress [7]. The high scores suggest that nurses tend to act courageously in ethical decision-making processes and are willing to take responsibility for protecting patient rights. Moral courage is of critical importance, especially for palliative care nurses. This is because nurses frequently face ethical dilemmas in palliative care and must advocate for what is right in these situations [24]. The shared decision-making process in palliative care represents an ethically complex challenge that demands moral courage [20]. Determining which interventions are non-beneficial or potentially unnecessary involves individual values and subjective judgments, often giving rise to ethical dilemmas. Moreover, the hierarchical nature of healthcare organizations can further complicate this process. The literature indicates that nurses sometimes feel compelled to follow physicians’ orders without question and may experience hesitation due to concerns about possible repercussions [25, 26]. Balancing the principles of justice, non-maleficence, and acting in the patient’s best interest is one of the core responsibilities of nurses. However, this responsibility must be carried out not only with clinical knowledge and skills but also with deep empathy and human sensitivity [4]. The high moral courage scores of palliative care nurses indicate that these nurses are strongly committed to professional ethical principles and have a strong motivation to make decisions in favor of patients.

A similar finding was noted in Rathert et al. (2016), where it was suggested that nurses with moral courage can reduce the moral conflicts around them, thereby reducing moral distress [27]. While moral courage is highly valued in nursing, it is widely recognized that demonstrating such courage is challenging due to factors like demanding work environments, role ambiguity, hierarchical power dynamics, institutional culture, and the unique characteristics of healthcare settings. Nurses who display moral courage may encounter resistance from colleagues or supervisors, intimidation, psychological and/or physical aggression, threats to their job security or professional standing, and various forms of obstruction [28].

The results of the study suggest that many individual and institutional factors can affect moral courage. Factors such as education level, whether the nurse chose the unit they work in, and their self-assessment of health ethics knowledge caused significant differences in moral courage scores. The significantly higher moral courage scores of nurses with a master’s degree compared to those with lower education levels indicate that the level of ethical knowledge and academic education can enhance moral courage. Previous studies also suggest that education level influences nurses’ moral courage [11, 29]. This may be attributed to the fact that nurses with higher education possess greater professional knowledge and enhanced judgment regarding patient treatment and care plans. Nurses who had the opportunity to choose their work unit scored significantly higher on moral courage compared to those who did not, suggesting that job satisfaction and motivation may play important roles in ethical decision-making processes. Education can bolster moral courage by increasing individuals’ awareness of ethical issues and enhancing their capacity to navigate ethical dilemmas. Furthermore, nurses working in their preferred units may experience better adaptation to the work environment and greater professional autonomy, factors that likely contribute to courageous behavior when confronting ethical challenges. Nurses who rated their knowledge of healthcare ethics as “excellent” demonstrated significantly higher moral courage scores, reinforcing the notion that ethical knowledge influences an individual’s confidence in moral decision-making. Moral courage is a fundamental component of nurses’ ethical competence and can be cultivated and developed over time [9]. Therefore, moral courage can be fostered through educational initiatives, interdisciplinary discussions, and supportive leadership models (Numminen, 2019). Furthermore, developing moral courage before entering the profession is crucial for nurses to become competent in ethical decision-making processes and demonstrate moral courage [30].

A positive correlation was found between nurses’ ages and their ethical courage scores in nursing. A study found a positive correlation between planned training given to nurse leaders and improvements in professional moral courage scale scores, as well as nurse management experience (up to 20 years) [31]. This may suggest that experience and professional development increase with age, which may lead to an increase in ethical courage. As nurses age, they encounter more clinical situations and become more competent in handling ethical dilemmas. Although the results in the current study were not statistically significant, those who felt they needed to show moral courage in nursing practice had higher moral courage scores than others.

The negative correlation between nurses’ number of night shifts per month and their moral courage suggests that excessive working conditions may cause nurses to be more cautious in their decision-making processes and perhaps less courageous. A study has shown that individual and organizational factors can hinder moral courage. Personality traits such as unwillingness, lack of responsibility, shyness, lack of sensitivity, insufficient motivation, and low self-confidence can prevent the development of moral courage [10]. Rakhshan et al. (2021) also accepted that lack of interest in the profession, lack of job motivation, moral silence, insufficient self-confidence, and egocentrism may be barriers to the formation of moral courage [32]. Intense shifts can increase nurses’ stress levels, which may make them feel less secure in making ethical decisions. Moreover, long working hours and excessive shifts may have negative effects on nurses’ physical and mental health, which may, in turn, negatively impact their moral courage.

### Limitations of the study

This study has some limitations. Firstly, since the research has a cross-sectional design, causal relationships between variables cannot be determined. Additionally, because the study was conducted solely through an online survey method, data based on participants’ self-reports were used. This may lead to social desirability bias. Another limitation of the study is that the sample is limited to nurses from a specific time period and geographic region. Therefore, the generalizability of the results to different cultural and institutional contexts should be carefully evaluated. In this study, the NMCS-TR used was found to have a unidimensional structure, as the original four-factor model could not be confirmed in the Turkish validation study. Therefore, analyses were conducted using only the total score, and a more detailed examination of the subdimensions was not possible. This limits the ability to fully explore the multidimensional nature of moral courage.

## Conclusions

The research findings indicate that palliative care nurses exhibit high levels of moral courage. Their propensity to make courageous decisions grounded in ethical principles offers a significant advantage in safeguarding patient rights and navigating ethical dilemmas. Moreover, factors such as educational attainment, choice of work unit, and self-assessed knowledge of healthcare ethics have been shown to meaningfully influence moral courage. Notably, nurses holding a master’s degree demonstrated higher moral courage scores, suggesting that ethical education and advanced academic training contribute to strengthening moral courage.

However, the results also highlight that working conditions and organizational dynamics can adversely affect moral courage. High workload intensity, frequent shifts, and insufficient institutional support may cause nurses to approach ethical decision-making with increased caution and hesitation. In this regard, implementing policies that promote professional autonomy, improving workplace conditions, and enhancing ethics education programs represent critical strategies for fostering moral courage.

Future research should aim to include larger and more diverse samples to explore the factors influencing moral courage in greater depth. Additionally, qualitative methods could provide deeper insights into ethical decision-making processes and the manifestations of moral courage. Further studies may also evaluate the effectiveness of targeted training programs designed to enhance moral courage and assess the impact of supportive institutional policies.

## Data Availability

Data are available upon request from the corresponding author.

## Declarations

### Constent to participate

Informed consent was obtained from all individual participants included in the study.
